# Methylglyoxal Impairs Mitochondrial Bioenergetics and Microglial Functions

**DOI:** 10.3390/cells15211950

**Published:** 2026-10-27

**Authors:** Taketo Taguchi, Pamela Maher

**Affiliations:** Department of Cellular Neurobiology, The Salk Institute for Biological Studies, La Jolla, CA 92037, USA

**Keywords:** Alzheimer’s disease, microglia, methylglyoxal, mitochondrial bioenergetics, phagocytosis, brain homeostasis

## Abstract

Aging is accompanied by a progressive disruption of brain homeostasis, yet the molecular mechanisms underlying this age-associated deterioration remain poorly understood. Microglia play a critical role in maintaining brain homeostasis by clearing cellular debris and neurotoxic protein aggregates. Here, we investigated the effects of methylglyoxal (MG), a highly reactive endogenous metabolite that accumulates with aging, on microglial functions using the murine microglial cell line BV2. We assessed the effects of MG exposure on multiple aspects of microglial biology in both control and bacterial lipopolysaccharide (LPS)-treated cells, including phagocytic activity, cellular proliferation, cytokine secretion, mitochondrial bioenergetics, mitochondrial membrane potential, and intracellular and mitochondrial reactive oxygen species. We found that MG significantly impaired mitochondrial bioenergetics, leading to reduced cellular energy production, and markedly suppressed multiple essential microglial functions, including phagocytosis. Given the high reactivity of MG with arginine, we also investigated whether arginine depletion contributes to these functional impairments. Arginine depletion recapitulated the impairment of mitochondrial bioenergetics and microglial function induced by MG, demonstrating that arginine availability is a critical determinant of microglial metabolic fitness and immune function. These findings suggest that the age-associated accumulation of MG impairs mitochondrial bioenergetics and microglial functions, thereby potentially accelerating neurodegenerative processes.

## 1. Introduction

Aging is the foundation of many diseases, and this foundation is continuously being built for several decades before a disease phenotype appears [1,2]. Alzheimer’s disease (AD) is one of these diseases and, as the worldwide population ages, it is predicted to increase further in the coming decades [3,4]. One hallmark of the aged brain is a progressive accumulation of waste and debris, such as metabolic waste, abnormal protein aggregates, uncleared pathogens and their remnants [5]. While clearance of such debris is efficient in young cells and tissues, aging leads to a decrease in the ability to clear debris [6]. Multiple mechanisms are cooperatively involved in removing debris from the brain including phagocytic clearance by the brain-resident immune cells, microglia. Microglia are crucial in maintaining the homeostasis of a healthy brain [7]. They actively survey the environment by extending processes, and once found, microglia remove debris by phagocytosis. In the aged brain, microglia seemingly function less actively, and the gradual accumulation of uncleared debris may help set the stage for future neurodegeneration [1]. We were interested in why microglia function less actively in the aging brain and propose a possible connection among the following three pathways: (1) energy production; (2) immunosuppression in microglia; and (3) a metabolic byproduct, methylglyoxal. Understanding why microglial function begins to fail as we age is a critical step to preventing age-associated cognitive decline, including AD.

**Figure 1:**
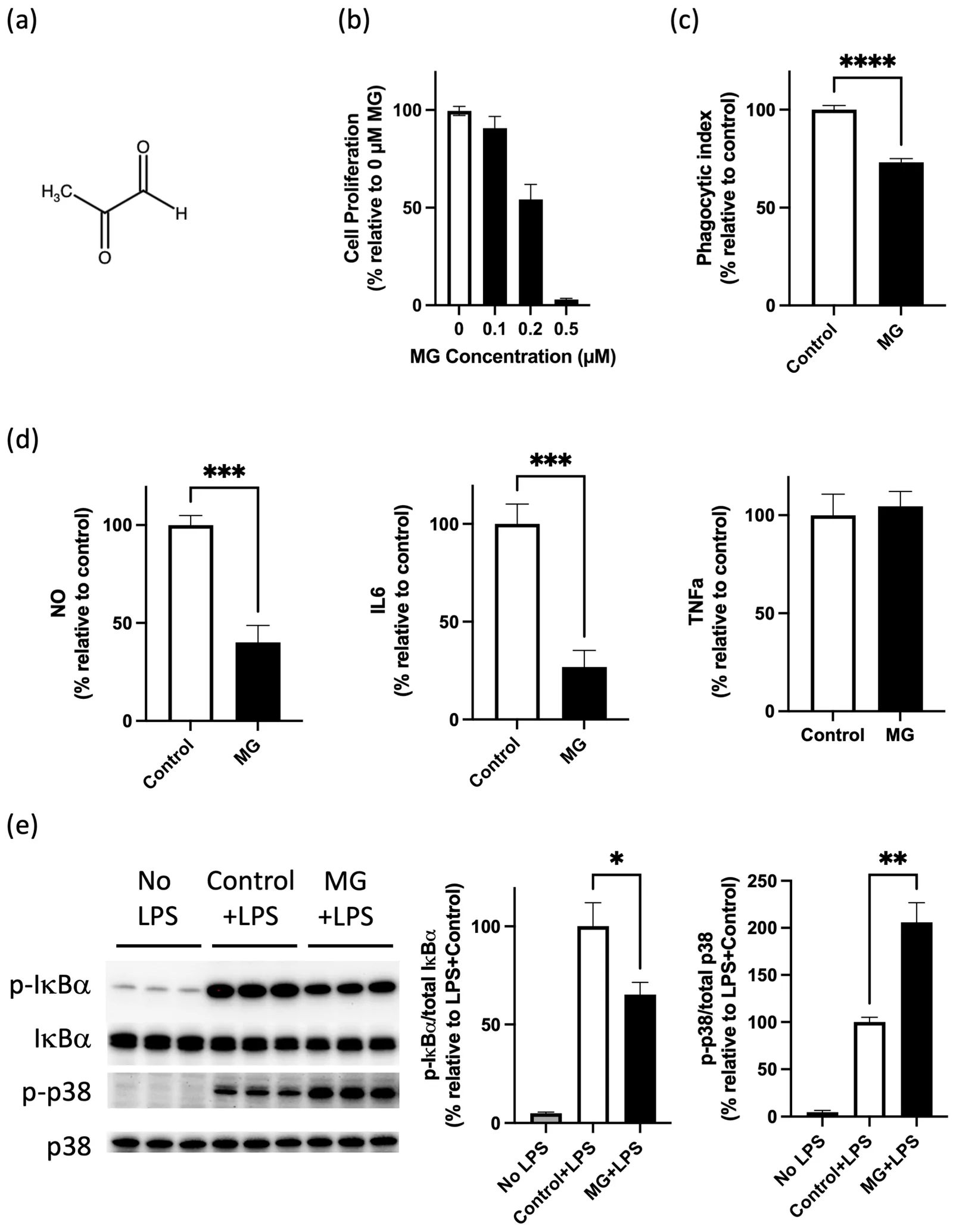
MG suppresses microglial functions. (**a**) Chemical structure of MG. (**b**) Proliferation assay. BV2 cells were treated with MG (100, 200 or 500 μM). After 24 h of treatment, the cell number was determined by the MTT assay and normalized to cells not treated with MG. (**c**) Phagocytosis assay. Pre-opsonized latex beads were added to the BV2 cells at a concentration of 10 beads per cell and incubated for 90 min. The cells were then harvested and analyzed by flow cytometry. (**d**) Nitric oxide and cytokine assays. BV2 cells were treated or untreated with 25 ng/mL LPS alone or in the presence of MG for 24 h and the cell culture supernatants assayed for NO production using the Griess assay and cytokine levels using specific ELISAs. (**e**) BV2 cells were treated or untreated with 25 ng/mL LPS alone or in the presence of MG for 24 h. Cell extracts were prepared and equal amounts of protein analyzed by SDS-PAGE and Western blotting. *p* values less than 0.05 were considered statistically significant (* *p <* 0.05, ** *p <* 0.01, *** *p <* 0.001, and **** *p <* 0.0001).

Our ideas about the mechanisms underlying these age-associated changes in microglia originated in studies where we investigated the association between type 2 diabetes (T2D) and dementia in animal models [8,9,10]. Importantly, diabetes is associated with accelerated brain aging [11]. Epidemiological studies have also shown that T2D patients are more likely to develop dementia [12]. In addition to the many comorbidities that T2D patients have, impairment of debris clearance in the brain is frequently noted [13]. T2D patients are known to abundantly produce a toxic glycating molecule called methylglyoxal (MG, Figure 1a) [14]. MG is known to initiate the production of advanced glycation end-products and protein aggregation. Higher concentrations of MG were reported to be present in the cerebrospinal fluid of AD patients as compared to healthy controls [15]. In addition, higher plasma MG levels were reported to correlate with faster cognitive declines in the elderly [16,17]. It is important to note that MG is an endogenous byproduct of glycolysis and is naturally and continuously produced even in healthy individuals, although T2D patients produce several-fold more MG than healthy individuals [14,18]. In this study, we show that MG significantly dampens mitochondrial energetics and suppresses microglial functions including phagocytosis. These results suggest that the age-associated gradual increase in MG in the brain could cause energy depletion and immunosuppression in microglia.

## 2. Materials and Methods

Methylglyoxal (MG) was purchased from VWR (Radnor, PA, USA, Cat# 0215555825). Bacterial lipopolysaccharide (LPS) was purchased from Sigma Aldrich (Saint Louis, MO, USA). MG was diluted in phosphate-buffered saline (PBS) and added to the culture medium at the indicated concentrations. LPS was dissolved in PBS and added to the culture medium at the indicated concentration. Control cells were left untreated. Seahorse XFe96 FluxPak (Cat# 102416) and the Seahorse XF Cell Mito Stress Test Kit (Cat# 103015) were from Agilent Technologies (Santa Clara, CA, USA). JC-10 Mitochondrial Membrane Potential Assay Kit (Cat# ab112134) was from Abcam (Burlingame, CA, USA). Pierce BCA Protein Assay Kit (Cat# 23227), Cell Lysis Buffer (Cat# FNN0011), SuperSignal West Pico PLUS Chemiluminescent Substrate (Cat# 34578), CellROX Green Reagent (Cat# C10444) and MitoSOX Red Mitochondrial Superoxide Indicator (Cat# M36008) were from Thermo Fisher Scientific (Carlsbad, CA, USA). Criterion XT Bis-Tris Protein Gradient Gel (Cat# 3450125), XT MOPS Running Buffer (Cat# 1610788), Trans-Blot Turbo RTA Transfer Kit (Cat# 1704275), Precision Plus Protein Standards (Cat# 1610373) were from Bio-Rad (Hercules, CA, USA). Protease/Phosphatase Inhibitor (Cat#5872S) was from Cell Signaling Technology (Danvers, MA, USA).

### 2.1. Cell Culture

Mouse BV2 microglial cells (obtained from Dr. Grace Y. Sun (University of Missouri at Columbia, MO, USA) were maintained in low-glucose (5 mM) DMEM (Thermo Fisher Scientific, Cat# 11885084) with 10% fetal bovine serum (Invitrogen, Carlsbad, CA, USA) and 1% antibiotics (Invitrogen, Cat# 10378016) at 10% CO_2_. For MG treatment, MG was used at a concentration of 200 μM, as previously reported in BV2 microglial cells [19]. For the arginine depletion experiments, SILAC medium (Invitrogen, Cat. No. A2493901) was supplemented with D-glucose, sodium pyruvate, L-glutamine, L-lysine and L-arginine in control conditions, whereas L-arginine was omitted from the medium for arginine-depleted conditions.

### 2.2. Proliferation Assay

BV2 cells were plated in 96 well plates at a density of 3 × 10^4^ cells in low-glucose DMEM. The next day the medium was exchanged for high-glucose (25 mM) DMEM and the cells were treated with LPS (25 ng/mL) and MG (100, 200 or 500 μM). After 24 h of treatment, cell number was estimated by the 3-(4,5-dimethylthiazol-2-yl)-2,5-diphenyltetrazolium bromide (MTT) assay, and normalized to non-treated wells. Results were confirmed by visual inspection of the wells. Eight wells per treatment condition were used for each independent experiment.

### 2.3. Phagocytosis Assay

BV2 cells were plated in 35 mm dishes at a density of 3 × 10^5^ cells in low-glucose DMEM. After overnight culture, the medium was changed to high-glucose (25 mM) DMEM with or without MG (200 μM) for 3 h. Pre-opsonized latex beads (L3030, 2 µm, Sigma Aldrich; in 50% FBS in PBS) were then added to the cells at a concentration of 10 beads per cell and incubated for 90 min. The cells were harvested using Trypsin (0.25%, Thermo Fisher Scientific) and washed three times with ice-cold FACS buffer (1% FBS and 0.4% sodium azide in PBS) and analyzed by flow cytometry (FACS Symphony A3, BD Biosciences, San Jose, CA, USA). Six samples per treatment (control vs. MG) per experiment were used for each independent experiment.

### 2.4. Nitric Oxide and Cytokine Assays

BV2 cells were plated in 35 mm dishes at a density of 5 × 10^5^ cells in low-glucose DMEM. The following day, the medium was replaced with high-glucose DMEM (25 mM glucose) for the MG treatment experiment and the cells were treated with LPS (25 ng/mL) in the presence or absence of MG (200 μM). For the arginine depletion experiments, the medium was replaced with SILAC medium supplemented with D-glucose, sodium pyruvate, L-glutamine, L-lysine, and with or without L-arginine and the cells were treated with LPS (25 ng/mL). After 24 h, the medium was collected and briefly centrifuged to eliminate floating cells. A 100 µL sample was then used to measure nitric oxide (NO) with the Griess Reagent (Sigma Aldrich) in a 96-well plate. After incubation for 10 min at room temperature, the absorbance at 550 nm was read on a SpectraMax M5 microplate reader (Molecular Devices, San Jose, CA, USA). The remaining culture supernatants were stored at −20 °C until used to determine their levels of IL6 and TNFα using ELISAs (R&D Systems, Minneapolis, MN, USA) according to the manufacturer’s instructions. The cells left in the dishes after removal of the culture supernatants were assayed for live cell number using the MTT assay, and NO or cytokine production was normalized to the MTT assay results. Three dishes per treatment condition were used for each independent experiment.

### 2.5. Western Blotting

For sample preparation [20], BV2 cells were plated in 35 mm dishes at a density of 5 × 10^5^ cells in low-glucose DMEM. The next day the medium was exchanged for high-glucose (25 mM) DMEM and the cells were treated with LPS (25 ng/mL) in the presence or absence of MG (200 μM). After 24 h, the cells were washed twice with ice-cold calcium- and magnesium-free PBS and collected by scraping into lysis buffer supplemented with 1× protease inhibitor cocktail and 1× phosphatase inhibitor cocktail. The lysates were kept on ice for 30 min, sonicated, and centrifuged to remove insoluble material. Protein concentrations were quantified by the BCA assay and adjusted to equal concentrations. 5× Western blot sample buffer (74 mM Tris-HCl, pH 8.0, 6.25% SDS, 10% β-mercaptoethanol, 20% glycerol) was then added to achieve a final buffer concentration of 2.5× and samples were boiled for 5 min. Equal amounts of cellular protein (10–20 μg per lane) were then separated by SDS-PAGE using 4–12% gradient Criterion XT Precast Bis-Tris Gels (Bio-Rad) and transferred onto PVDF membranes with a Trans-Blot Turbo System (Bio-Rad). Membranes were blocked with 5% skim milk in TBST (20 mM Tris buffer, pH 7.5, 0.5 M NaCl, 0.1% Tween20) for 1 h at room temperature, followed by overnight incubation at 4 °C with primary antibodies diluted in 5% BSA in TBST. The primary antibodies used were from Cell Signaling Technology: phospho IκBα (Cat# 4812, 1:1000), IκBα (Cat# 2859, 1:1000), phospho p38 (Cat# 4511, 1:1000), p38 (Cat# 8690, 1:1000). After washing with TBST, the membranes were incubated for 1 h at room temperature with horseradish peroxidase (HRP)-conjugated goat anti-rabbit secondary antibodies (Bio-Rad) diluted 1:5000 in 5% skim milk in TBST. After additional washing, protein bands were visualized using SuperSignal West Pico PLUS Chemiluminescent Substrate (Thermo Fisher Scientific) and imaged using a ChemiDoc MP Imaging System (Bio-Rad). Band intensities were quantified using Image Lab software (Version 6.1.0, Bio-Rad). Each Western blot was performed three times with independent protein samples.

### 2.6. Seahorse XF Analysis

The assay was carried out using a Seahorse XFe96 Extracellular Flux Analyzer with a XF Cell Mito Stress Test Kit (Seahorse Bioscience, North Billerica, MA, USA) as described previously [21]. Briefly, BV2 cells were seeded at 1 × 10^4^ cells/well onto the Seahorse XFe96 plates in low-glucose DMEM media supplemented with 10% fetal bovine serum overnight. The next day, for MG treatment experiments, the media was replaced with complete Seahorse XF DMEM assay medium supplemented with 10 mM glucose, 1 mM pyruvate and 2 mM L-glutamine, at pH 7.4, with or without MG (200 μM). For the arginine depletion experiments, the medium was replaced with the SILAC medium supplemented with D-glucose, sodium pyruvate, L-glutamine, L-lysine, with or without L-arginine. The cells were incubated for 24 h prior to the XF Cell Mito Stress tests according to the manufacturer’s instructions. Mitochondrial ETC inhibitors were used at the following concentrations: 1.5 μM oligomycin (Oligo), 2 μM FCCP, and 0.5 μM of a 1:1 mixture of rotenone (Rot) and antimycin A (AA). Data were normalized to total protein as determined by the BCA assay/well. Each treatment condition was analyzed in 6–24 wells per independent experiment.

### 2.7. Mitochondrial Membrane Potential Assay

The assay procedure was previously described [21]. Briefly, BV2 cells were seeded at 1.5 × 10^4^ cells/well onto 96 well plates in low-glucose DMEM. The next day, for MG treatment, the medium was exchanged for high-glucose DMEM (25 mM glucose) and the cells were treated with LPS (25 ng/mL) in the presence or absence of MG (200 μM). For the arginine depletion experiments, the medium was replaced with the SILAC medium supplemented with D-glucose, sodium pyruvate, L-glutamine, L-lysine, with or without L-arginine and the cells were treated with LPS (25 ng/mL). After 2 h, the cells were subjected to a JC-10 mitochondrial membrane potential assay according to the manufacturer’s procedure. Fluorescence intensities (Ex/Em = 490/525 nm, and Ex/Em = 540/590 nm) of each well were measured on a SpectraMax M5 microplate reader. The ratio of fluorescence intensity (590/525 nm) was used to determine the mitochondrial membrane potential. Increasing ratios indicate mitochondrial membrane hyperpolarization. Results were verified by live-cell imaging under a fluorescence microscope. Each condition was analyzed in six wells per independent experiment.

### 2.8. Reactive Oxygen Species (ROS) Measurement

The assay procedure was previously described [21]. BV2 cells were seeded at 1 × 10^4^ cells/well onto 96 well plates. The next day, for MG treatment, the medium was exchanged for high-glucose DMEM (25 mM glucose) and the cells were treated with LPS (25 ng/mL) in the presence or absence of MG (200 μM). For the arginine depletion experiments, the medium was replaced with the SILAC medium supplemented with D-glucose, sodium pyruvate, L-glutamine, L-lysine, with or without L-arginine and treated with LPS (25 ng/mL). After 24 h, whole-cell ROS and mitochondrial superoxide ROS were detected with CellROX Green reagent (Ex/Em = 485/520 nm) and MitoSOX Red reagent (Ex/Em = 510/580 nm), respectively. Experiments were performed according to the manufacturer’s instructions. Fluorescence was measured on a SpectraMax M5 microplate reader. Data were normalized to total protein as determined by the BCA assay. Each treatment condition was analyzed in eight wells per independent experiment.

### 2.9. Statistical Analysis

Data were collected from at least three independent experiments. Analyses were performed using GraphPad Prism 10. The data were analyzed by Student’s *t*-test and are presented as the mean ± SD. *p* values less than 0.05 were considered statistically significant (* *p <* 0.05, ** *p <* 0.01, *** *p <* 0.001, and **** *p <* 0.0001).

## 3. Results

### 3.1. MG Suppresses Multiple Microglial Functions

To determine a working concentration of methylglyoxal (MG) for subsequent experiments, we first performed a concentration titration using a proliferation assay in BV2 microglial cells. After 24 h of MG treatment at 200 μM, cell proliferation as estimated by the MTT assay was approximately 50% of that of untreated cells, whereas no detectable MTT reduction was observed at 500 μM, suggesting substantial cytotoxicity at this high concentration (Figure 1b). Consistent with these results, untreated cells showed clear proliferation during the 24 h incubation period, whereas the number of cells treated with 200 μM MG appeared largely unchanged and no morphological alterations indicative of cytotoxicity were noted. At 500 μM MG, cells exhibited marked morphological changes, including cell rounding and detachment, consistent with substantial cytotoxicity. It is important to note that the decrease in cell proliferation caused by MG treatment at 200 μM is reversible. When the MG-containing medium was replaced with MG-free medium, cell proliferation was restored. Based on these results, and consistent with a previous study using 200 μM MG in BV2 microglial cells [19], we selected 200 μM MG for subsequent experiments.

Because microglial proliferation is an important aspect of the response to infection, brain injury, and other forms of stimulation [22], we next asked whether MG also affects other important microglial functions. The removal of harmful debris from the brain is crucial for maintaining brain health [23], and timely phagocytosis of cellular debris is one of the key functions of microglia [24]. To examine whether the presence of MG changes microglial phagocytic properties, we treated BV2 cells for 3 h with 200 µM MG and then performed the bead uptake assay for 90 min using fluorescent latex beads in BV2 microglial cells. As shown in Figure 1c, FACS analysis of the cells showed that when MG was present in the culture medium, the capacity of BV2 cells to phagocytose the beads was significantly reduced.

Another important function of microglia is to secrete cytokines and other molecules in response to pathological cues. We thus examined the culture supernatants of BV2 cells following 24 h of LPS treatment for the presence of nitric oxide (NO) and the cytokines TNFα and IL-6. LPS treatment induced robust secretion of NO, as assessed using the Griess reagent. However, as shown in Figure 1d, NO production was significantly reduced in the presence of MG. LPS stimulation also markedly increased the secretion of IL-6 and TNFα, as measured by ELISA. However, when MG was present, the secretion of IL-6 decreased significantly. In contrast, the LPS-induced secretion of TNFα did not change in the presence of MG. These observations suggest that MG affects signaling pathways leading to NO production and IL-6 secretion, but not TNFα secretion. Thus, we examined the effects of MG on signaling pathways associated with cytokine secretion in LPS-stimulated BV2 cells by Western blot analysis. As shown in Figure 1e, when BV2 cells were stimulated with LPS, phosphorylation of IκBα significantly increased, indicating that the NF-κB signaling pathway was activated by LPS treatment. When MG was present, phosphorylation of IκBα decreased significantly, suggesting that activation of the NF-κB signaling pathway was reduced by MG treatment. LPS stimulation also increased phosphorylation of p38, showing that MAPK pathways were activated by LPS treatment. When MG was present, phosphorylation of p38 further increased. Overall, we found that when MG is present in the environment, key microglial functions are significantly altered.

### 3.2. MG Reduces Mitochondrial ATP Production

**Figure 2:**
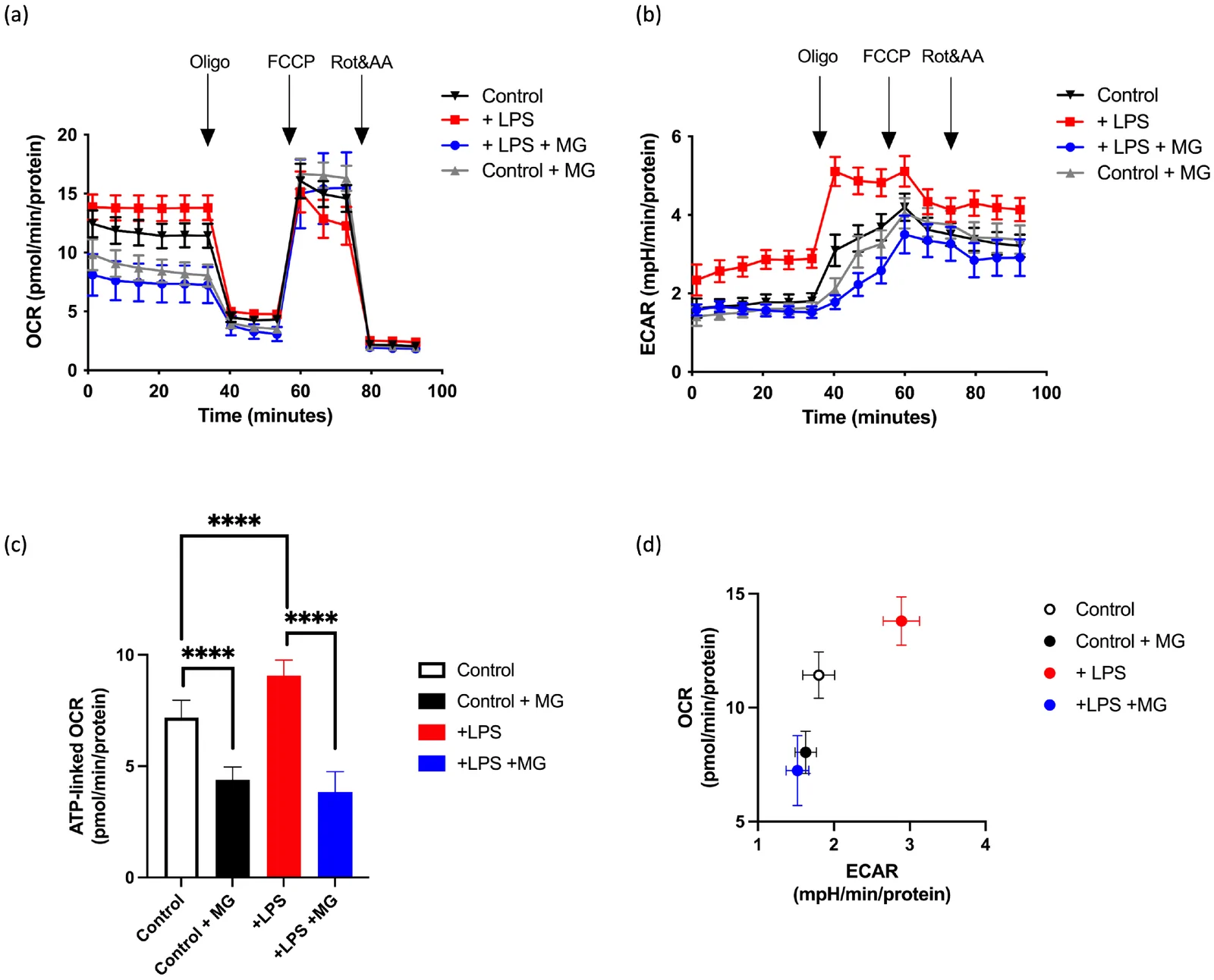
MG reduces mitochondrial energy production. (**a**) Mitochondrial oxygen consumption rate (OCR) profiles in BV2 cells after different treatments. Data were normalized to total protein/well. (**b**) Mitochondrial extracellular acidification rate (ECAR) profiles in BV2 cells after different treatments. Data were normalized to total protein/well. (**c**) ATP production in BV2 cells. *p* values less than 0.05 were considered statistically significant (**** *p <* 0.0001). (**d**) The energy phenotype profile of BV2 cells after different treatments. The LPS-stimulated cells changed to a more energetic phenotype while the MG-treated cells changed to a more quiescent phenotype, even when stimulated with LPS.

Next, we investigated if the observed suppression of microglial function is accompanied by any changes in energy production that are required to sustain necessary immune reactions [25]. For these studies, we used the Seahorse mitochondrial stress test to examine the oxygen consumption rate (OCR) and extracellular acidification rate (ECAR) (Figure 2a,b). When BV2 cells were stimulated with LPS for 24 h, OCR and especially ECAR increased. It has been shown previously that LPS stimulation shifts microglial energy production from OXPHOS to glycolysis [26]. However, in the presence of MG, both OCR and ECAR were markedly reduced. Importantly, ATP-linked OCR was significantly reduced in the presence of MG (Figure 2c). Figure 2d shows the cell energy phenotype profile: cells in the upper right quadrant are more energetic; cells in the lower left quadrant are more quiescent; cells in the upper left quadrant are more aerobic; cells in the lower right quadrant are more glycolytic. As expected, the LPS-stimulated cells changed to a more energetic phenotype to meet the energy requirements for necessary immune reactions. However, when MG was in the medium, the cell energy phenotype was shifted to a more quiescent state even though the cells were stimulated with LPS.

### 3.3. Arginine Depletion Causes Microglial Immunosuppression

**Figure 3:**
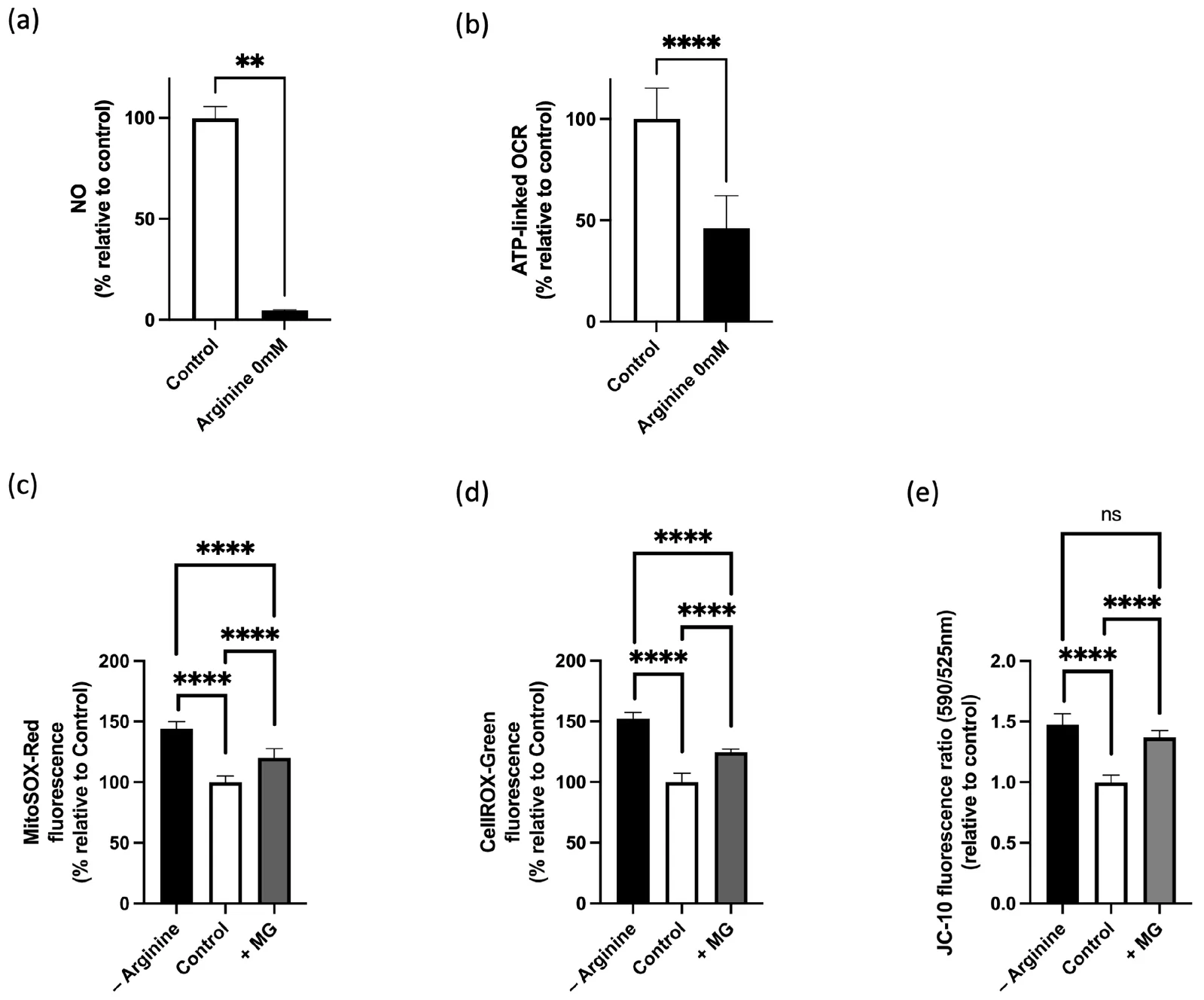
Arginine depletion causes microglial immunosuppression. (**a**) Nitric oxide assay. BV2 cells grown in control or arginine-depleted medium were treated or untreated with 25 ng/mL LPS alone or in the presence of MG for 24 h and the cell culture supernatants assayed for NO production using the Griess assay. (**b**) ATP production in BV2 cells. Mitochondrial oxygen consumption rate (OCR) profiles of BV2 cells grown in control or arginine-depleted medium were studied. Data were normalized to control (with arginine). (**c**) Mitochondrial membrane potential of BV2 cells grown in control, arginine-depleted or control medium with 200 µM MG for 2 h. Data were normalized to control. (**d**) Cellular ROS (CellROX) levels of BV2 cells grown in control, arginine-depleted or control medium with 200 µM MG for 24 h. Data were normalized to control. (**e**) Mitochondrial ROS (MitoSOX) levels of BV2 cells grown in control, arginine-depleted or control medium with 200 µM MG for 24 h. Data were normalized to control. *p* values less than 0.05 were considered statistically significant (** *p <* 0.01, **** *p <* 0.0001, and ns, not significant).

The next question was: How is MG inducing these changes in microglia? Since MG is a highly reactive aldehyde, especially toward arginine residues, we hypothesized that free arginine might be immediately consumed by MG and that this consumption results in a shortage of arginine in the local environment. Since arginine is required for NO production [22], we first studied NO production in BV2 cells cultured in arginine-free media for 24 h. As expected, LPS-induced NO production was not observed when arginine was depleted from the culture medium (Figure 3a). The Seahorse mitochondrial stress test analysis also revealed that 24 h of arginine depletion significantly suppressed ATP production (Figure 3b).

### 3.4. Both the Presence of MG and the Absence of Arginine Increase Mitochondrial Membrane Potential and Reactive Oxygen Species Production

Since we found that both the presence of MG and the absence of arginine suppressed ATP production, we examined whether or not they influence the mitochondrial membrane potential that serves as the driving force for ATP synthesis by ATP synthase. When MG was present or arginine was absent in the culture medium for 2 h, the mitochondrial membrane potential, assessed using JC-10, significantly increased (hyperpolarized) by 37% and 48%, respectively (Figure 3c). Since an increase in mitochondrial membrane potential can increase ROS production, we also examined if the presence of MG or the absence of arginine increased ROS production. We studied ROS production using two different fluorogenic probes, CellROX and MitoSOX, in BV2 cells. The CellROX reagent is universal for cellular ROS detection, whereas the MitoSOX reagent is a ROS superoxide indicator specifically targeting mitochondrial ROS in live cells. As shown in Figure 3d, when MG was present or arginine was absent in the environment for 24 h, both cellular oxidative stress (CellROX) and mitochondrial superoxide (mitoSOX) production significantly increased.

## 4. Discussion

Microglia constantly survey the brain environment to help preserve brain homeostasis [7]. However, with advancing age, the maintenance of brain homeostasis gradually deteriorates, leading to problems such as the accumulation of uncleared debris [5]. Understanding what age-related changes lead to the impairment of surveillance and maintenance of homeostasis by microglia is an important question that remains to be fully addressed. Among the pathophysiological changes that occur during aging are increases in blood glucose levels and the development of insulin resistance [27,28]. These changes typically occur after middle age, and, in extreme cases, individuals develop T2D. In this project, we studied if and how MG, a toxic metabolite whose production is increased with age and exacerbated in T2D patients, affects microglial functions [29]. We show that MG can suppress mitochondrial energy production as well as multiple critical microglial functions. When MG is present, mitochondria are significantly hindered in their ability to generate ATP, even when stimulated to produce more energy, such as in response to LPS treatment. Instead of bolstering up OXPHOS and glycolysis in response to LPS, mitochondria stay quiescent, almost completely unresponsive to the stimulus. Such dampening of mitochondrial energy production is likely detrimental to appropriate microglial responses to immune stimuli. Indeed, we showed that the presence of MG also impairs microglial phagocytosis and proliferation. Diabetic patients are known to have less active phagocytic cells and a lower resistance to infection as compared with non-diabetic subjects [30]. Our results suggest that the functions of microglia in the brains of diabetic patients might also be weakened, making these patients more vulnerable to pathogenic insults and contributing to earlier development of dementia.

It is important to note that MG is also produced in healthy individuals because MG is a naturally occurring byproduct of glycolysis: about 0.1% of glycolytic flux is converted to MG [31]. The quantity of MG production is substantial when considered in relation to the amount of glucose typically consumed, and thus our body is continually at risk of being attacked by this massive amount of a highly reactive chemical. Fortunately, MG is typically detoxified through the glyoxalase system [32]. However, the activity of the glyoxalase system is known to decline with age, especially after middle age, indicating a possibility that the harmful effects of MG in the brain begin to arise after middle age [33]. Thus, we propose that after middle age, owing to the increasing amount of MG, microglial functions can become compromised, and the resulting immunosuppression may help to pave the way for the development of neurodegenerative diseases in the following decades. For T2D patients, the harmful effect of MG may arise earlier and in a greater magnitude [34].

Connections between MG and immunosuppression have been suggested by several previous studies. For example, in sepsis, a massive amount of MG is produced, and immune functions are suppressed [35,36]. The suppression of both OXPHOS and glycolysis was seen in “immune paralysis” of sepsis [35,37]. In cancer, Baumann et al. reported that MG produced by nearby myeloid-derived suppressor cells reduced both OXPHOS and glycolysis in T cells and dampened T cell functions against cancer cells [38]. Price et al. also showed that MG suppresses T-cell proliferation and cytokine production [39]. These previous findings in adaptive immune cells mirror our findings in innate immune cells: MG can suppress microglial energy production and immune functions including proliferation, cytokine production and phagocytosis.

It is worth considering that the suppression of immune responses by MG could, under certain circumstances, be beneficial. LPS can elicit a strong inflammatory response in microglia, and excessive or sustained neuroinflammation may contribute to neuronal damage. Thus, attenuation of LPS-induced inflammatory responses does not necessarily indicate a detrimental effect. However, an effective microglial response is also important for host defense and the maintenance of brain homeostasis, and excessive suppression could impair these functions. In our study, MG reduced mitochondrial function and cellular energy production, accompanied by impaired microglial phagocytosis and reduced responsiveness to inflammatory stimuli. These findings suggest that the effects of MG extend beyond the attenuation of potentially harmful inflammation and rather reflect a broader impairment of microglial functions. Therefore, although MG-mediated suppression of inflammation could be beneficial in some contexts, the accompanying loss of metabolic capacity and essential microglial functions may limit this benefit, particularly in the context of aging, when the accumulation of cellular damage requiring an effective microglial response may increase.

The biochemical effects of MG are not yet fully understood. MG is a highly reactive chemical that is known to react with various biomolecules, e.g., proteins, nucleic acids and lipids. The modified biomolecules can both show a decrease in their normal functions and form aggregates. The observed immunosuppression in this study could be caused, at least in part, by reactions of MG with these biomolecules. Another possible mechanism that could contribute to the immunosuppression caused by MG is the direct consumption of free arginine by MG. We showed that arginine depletion phenocopied many of the effects of MG, including suppression of mitochondrial ATP production, mitochondrial membrane hyperpolarization, and increased ROS production. Connections between arginine depletion and immunosuppression have also been reported in some previous studies. For example, Baumann et al. showed that the MG-induced reduction in T cell activity is mediated by arginine depletion [38]. Similarly, we suggest that the increase in MG in the brain might deplete arginine and contribute to the impairment of microglial functions.

What are the sources of MG in the brain? The brain is a highly energy-demanding organ and heavily depends on glucose as an energy source. Thus, brain cells will continuously produce MG endogenously. Among brain cell types, astrocytes are known to be more glycolytic than others [40] and also have high glyoxalase activity [41] which is necessary for astrocytes to detoxify the MG that results from high levels of glycolysis. However, as glyoxalase activity declines after middle age, there could be an increase in MG leakage from astrocytes leading to the suppression of multiple microglial functions [33,42]. Microglia themselves could also be a source of MG, at least under pro-inflammatory conditions. Wei et al. showed that when microglia were activated by LPS, MG accumulated in the microglia [19]. This suggests that under pro-inflammatory conditions, microglia might suppress their own activity through MG that they themselves produce.

A limitation of our study relates to the concentration of MG used especially as CSF levels were found to be 10–20 µM [15] while cellular free MG levels were estimated to be in the low micromolar range [43]. However, MG is highly reactive and has a very short half-life both in complete culture medium and in cells [44] such that the local concentration is likely significantly higher [45]. Moreover, it has been estimated that more than 90% of cellular MG is bound to macromolecules so that actual levels of MG can reach up to 300 μM if bound MG is taken into consideration [46,47]. Furthermore, the concentration of 200 µM MG used in our study is well within the range used in many previous studies [41,48,49,50,51].

Finally, although our study primarily focused on the effects of MG on microglial function, our findings also raise the possibility that the age-associated increase in MG and the resulting decrease in NO production influences cerebral blood flow (CBF) and the regulation of neurovascular coupling (NVC). The importance of CBF regulation has attracted increasing attention, as its impairment may precede cognitive decline [52]. Spatial and temporal control of blood flow that meets energetic requirements is critical for proper brain functions. Vasodilators, especially nitric oxide (NO), play important roles connecting neuronal activities and local CBF. An increase in MG, especially after middle-age, and the resulting decrease in NO production could hinder proper NVC. Indeed, T2D patients are known to have decreased CBF and impaired NVC [53,54].

In conclusion, in this paper we report that the glycolysis metabolite, MG, dampens mitochondrial bioenergetics and impairs normal microglial functions including phagocytosis, cytokine secretion, and proliferation. We propose that rising MG levels in the brain after midlife progressively compromise microglial functions, especially in the context of T2D, allowing pathogenic processes to develop and spread. This may establish conditions that, over time, help foster the onset of neurodegenerative diseases such as AD.

## Data Availability

Data will be made available to interested researchers upon request.
